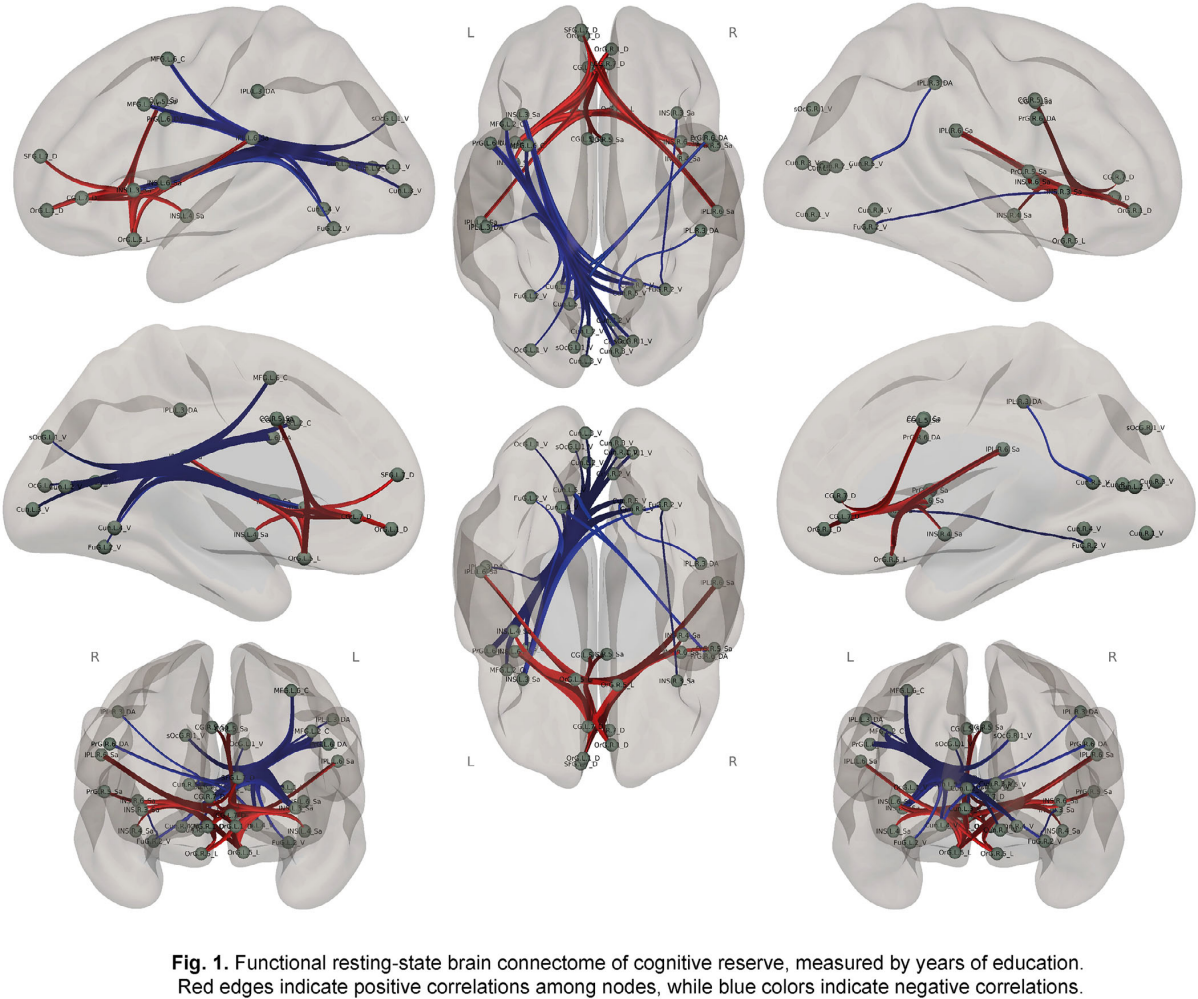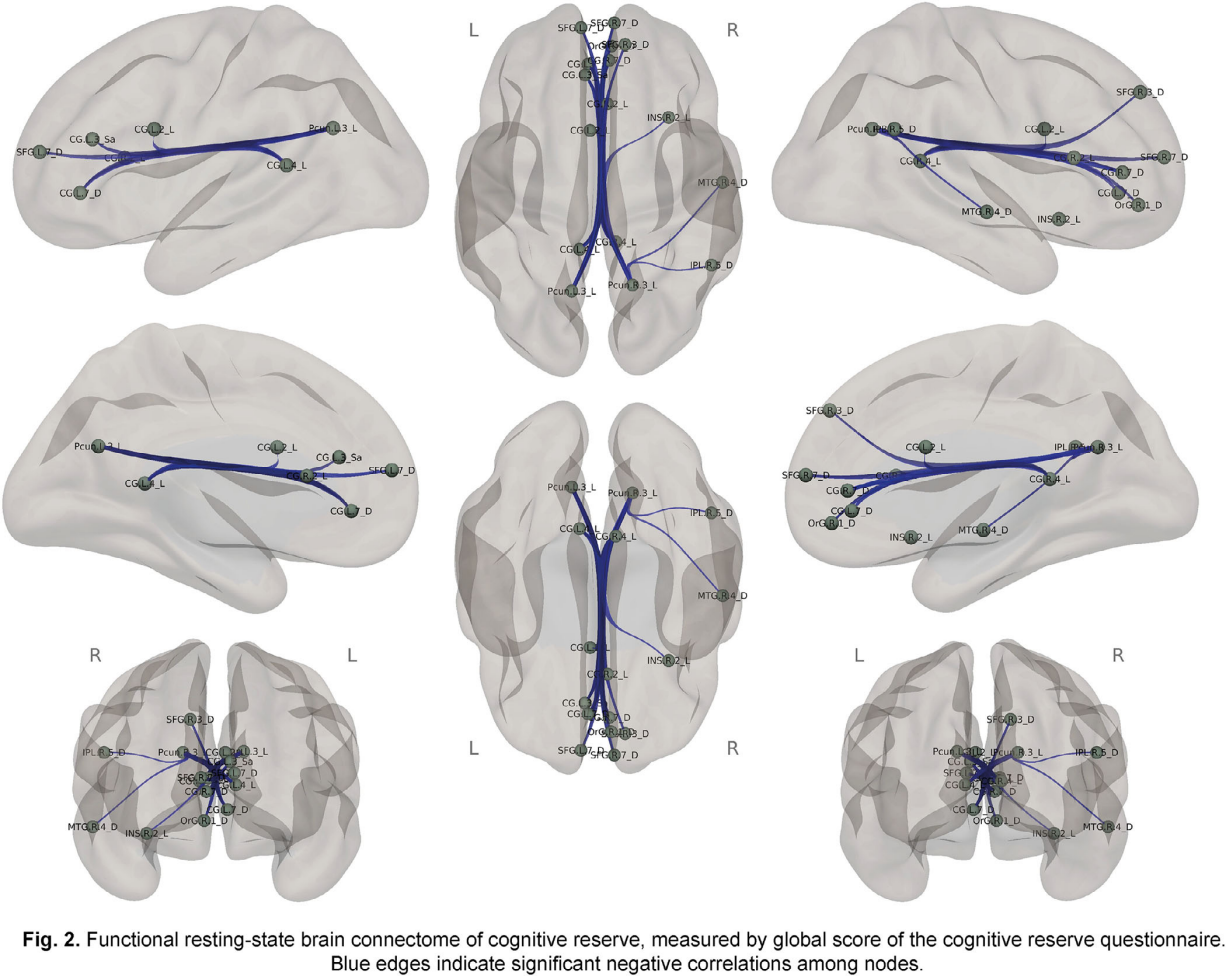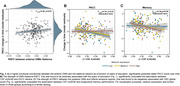# Cognitive reserve is associated with the recruitment of compensatory brain networks in individuals at risk for Alzheimer’s disease

**DOI:** 10.1002/alz.089176

**Published:** 2025-01-09

**Authors:** Aldana Lizarraga, Michalis Kassinopoulos, José María González‐de‐Echávarri, Jordi Huguet, Gonzalo Sánchez‐Benavides, Anna Brugulat‐Serrat, Marc Suarez‐Calvet, Marta Milà‐Alomà, Kaj Blennow, Henrik Zetterberg, Gwendlyn Kollmorgen, Clara Quijano‐Rubio, Jose Luis Molinuevo, Juan Domingo Gispert, Raffaele Cacciaglia, ALFA study

**Affiliations:** ^1^ Barcelonaβeta Brain Research Center (BBRC), Pasqual Maragall Foundation, Barcelona Spain; ^2^ Hospital del Mar Medical Research Institute (IMIM), Barcelona Spain; ^3^ Centro de Investigación Biomédica en Red de Fragilidad y Envejecimiento Saludable (CIBERFES), Madrid Spain; ^4^ Barcelonaβeta Brain Research Center (BBRC), Barcelona Spain; ^5^ Hospital del Mar Research Institute (IMIM), Barcelona Spain; ^6^ Servei de Neurologia, Hospital del Mar, Barcelona Spain; ^7^ Department of Veterans Affairs Medical Center, Northern California Institute for Research and Education (NCIRE), San Francisco, CA USA; ^8^ Clinical Neurochemistry Laboratory Sahlgrenska University Hospital, Mölndal Sweden; ^9^ Department of Psychiatry and Neurochemistry, Institute of Neuroscience and Physiology, University of Gothenburg, Mölndal Sweden; ^10^ Department of Psychiatry and Neurochemistry, Institute of Neuroscience and Physiology, The Sahlgrenska Academy at the University of Gothenburg, Mölndal Sweden; ^11^ Hong Kong Center for Neurodegenerative Diseases, Hong Kong China; ^12^ Department of Neurodegenerative Disease, UCL Queen Square Institute of Neurology, University College London, London, ‐ UK; ^13^ Wisconsin Alzheimer's Disease Research Center, School of Medicine and Public Health, University of Wisconsin‐Madison, Madison, WI USA; ^14^ Institute of Neuroscience and Physiology, University of Gothenburg, Mölndal Sweden; ^15^ UK Dementia Research Institute at UCL, London UK; ^16^ Roche Diagnostics GmbH, Penzberg Germany; ^17^ Roche Diagnostics International Ltd., Rotkreuz Switzerland; ^18^ Lundbeck A/S, Copenhagen Denmark; ^19^ Hospital del Mar Research Institute, Barcelona, Barcelona Spain; ^20^ Universitat Pompeu Fabra, Barcelona Spain; ^21^ Centro de Investigación Biomédica en Red de Bioingeniería, Biomateriales y Nanomedicina (CIBER‐BBN), Madrid Spain

## Abstract

**Background:**

Cognitive Reserve (CR) refers to the brain's ability to maintain optimal cognitive function despite damage or pathology. The neural implementation of CR is a major research focus, and resting‐state functional connectivity (RSFC) has emerged as a promising imaging correlate of CR. We assessed RSFC as a function of two different proxy measures of CR and further assessed the impact of these brain networks on longitudinal cognitive performance in a sample of cognitively unimpaired (CU) individuals at risk of Alzheimer's disease (AD).

**Method:**

Analyses were conducted in 328 CU individuals from the ALFA cohort (mean age=60.8, SD=4.74) with available CSF Aβ, p‐tau, resting‐state fMRI and longitudinal cognitive assessment (average follow‐up time=3.35 years, SD=0.53). CSF Aβ42 and Aβ40 were assessed with the exploratory NeuroToolKit, while p‐tau181 was measured with the Elecsys® Phospho‐Tau (181P) CSF immunoassay (both Roche Diagnostics International Ltd). We examined the impact of years of education (YOE) and global score of the Cognitive Reserve Questionnaire (CRQ) on the RSFC amongst 246 brain regions of the Brainnetome atlas using the CONN toolbox, selecting a cluster threshold of p<0.005, and adjusting or the effects of age, sex, and APOE status.

**Result:**

Of the entire sample, 38.4% had positive CSF Aβ42/40 markers. YOE was related to an increased RSFC between regions of the salience network and the anterior default‐mode network (DMN). Increased negative RSFC was found as a function of YOE between primary visual areas and regions of the executive control as well as dorsal attention (Figure 1). In models adjusted by CSF biomarkers, the increased RSFC between the anterior DMN and salience regions predicted better PACC score over time and further modulated the association between CSF Aβ42/40 and longitudinal PACC (Figure 2). CRQ score was associated with a decreased RSFC between the posterior DMN and inferior temporal areas. These patterns of reduced RSFC significantly mediated the impact of CSF Aβ42/40 on longitudinal memory performance (Figure 3).

**Conclusion:**

RSFC may provide insights into the mechanisms relating CR and cognitive resilience in preclinical AD. Further, the expression of RSFC patterns may serve as an outcome in intervention studies aiming to boost CR.